# Characterization of the Hepatic Transcriptome for Divergent Immune-Responding Sheep Following Natural Exposure to Gastrointestinal Nematodes

**DOI:** 10.3390/genes15060713

**Published:** 2024-05-30

**Authors:** Olivia Willoughby, Niel A. Karrow, Samla Marques Freire Cunha, Victoria Asselstine, Bonnie A. Mallard, Ángela Cánovas

**Affiliations:** 1 Centre for Genetic Improvement of Livestock, Department of Animal Biosciences, Ontario Agriculture College, University of Guelph, 50 Stone Road E, Guelph, ON N1G 2W1, Canada; owilloug@uoguelph.ca (O.W.); nkarrow@uoguelph.ca (N.A.K.); scunha@uoguelph.ca (S.M.F.C.); vasselst@uoguelph.ca (V.A.); 2 Department of Pathobiology, Ontario Veterinary College, University of Guelph, 50 Stone Road E, Guelph, ON N1G 2W1, Canada; bmallard@ovc.uoguelph.ca

**Keywords:** sheep, RNA-sequencing, immune response, gastrointestinal nematode

## Abstract

Infections with gastrointestinal nematodes (GINs) reduce the economic efficiency of sheep operations and compromise animal welfare. Understanding the host’s response to GIN infection can help producers identify animals that are naturally resistant to infection. The objective of this study was to characterize the hepatic transcriptome of sheep that had been naturally exposed to GIN parasites. The hepatic transcriptome was studied using RNA-Sequencing technology in animals characterized as high (*n* = 5) or medium (*n* = 6) based on their innate immune acute-phase (AP) response phenotype compared with uninfected controls (*n* = 4), and with biased antibody-mediated (AbMR, *n* = 5) or cell-mediated (CMR, *n* = 5) adaptive immune responsiveness compared to uninfected controls (*n* = 3). Following the assessment of sheep selected for innate responses, 0, 136, and 167 genes were differentially expressed (DE) between high- and medium-responding animals, high-responding and uninfected control animals, and medium-responding and uninfected control animals, respectively (false discovery rate (FDR) < 0.05, and fold change |FC| > 2). When adaptive immune responses were assessed, 0, 53, and 57 genes were DE between antibody- and cell-biased animals, antibody-biased and uninfected control animals, and cell-biased and uninfected control animals, respectively (FDR < 0.05, |FC| > 2). Functional analyses identified enriched gene ontology (GO) terms and metabolic pathways related to the innate immune response and energy metabolism. Six functional candidate genes were identified for further functional and validation studies to better understand the underlying biological mechanisms of host responses to GINs. These, in turn, can potentially help improve decision making and management practices to increase the overall host immune response to GIN infection.

## 1. Introduction

Gastrointestinal nematode (GIN) parasitism is a major production constraint for sheep producers worldwide. Infection with GINs can cause decreased production efficiency and poor animal welfare [1,2]. The most common GIN species in Ontario, CAN, include *Haemonchus contortus*, *Teladorsagia circumcincta*, and *Tricholostrongylus* spp. [3]. A mixed infection with these species is common in Ontario [3], which is home to approximately 1/3 of the national sheep flock in Canada (261,400/828,300 head) [4], though the most prevalent species can vary based on location. The most pathogenic GIN that affects grazing sheep is *H. contortus*; when significant burdens are present, infection causes clinical signs, including mild-to-severe anemia, hypoproteinemia, and possibly death [5]. Infection with *Tricholostrongylus* spp. is associated with gastrointestinal tract damage from their migration within the tissues of the host, enteritis, and watery diarrhea [6]. Infection with *T. circumcincta* causes diarrhea and ill thrift, as well as poor production [6]. Producers that have flocks with high levels of GIN infection suffer economic losses both from reduced herd productivity and the cost of treating and preventing infections [7].

The longstanding industry practice for managing GIN infection in grazing sheep has been routine anthelmintic treatment; however, this practice has led to the progressive loss of efficacy of most major classes of anthelmintics as parasite populations have adapted through selection pressure to evade their anthelmintic activities [8,9]. In 2013, Falzon et al. [8] found the resistance of GINs to ivermectin, fenbendazole, and levamisole to be 97% (28/29), 95% (19/20) and 6% (1/17) on the farms tested in their study, respectively. In addition to the costs incurred from anthelmintic use, sheep producers may also lose money due to clinically infected flocks being less productive [6,9,10,11,12]. It is estimated that inefficient helminth management (i.e., relying on an ineffective anthelmintic) decreases the carcass value of lambs by 10–14% [13,14]. In 1995, MacLeod [15] estimated that helminth infections in Australian sheep cost producers over AUD 222 million every year. In 2020, ref. [16] estimated the cost of GIN infection in Europe to be EUR 1.8 billion per year, with the majority of this cost coming from loss of production due to infection. Thus, GIN parasitism in North American sheep flocks can be inferred to have significant economic impacts as well. Further pressure is placed on sheep producers who rely on anthelmintics by consumers, as there is increasing interest in sourcing dietary protein from producers who do not use pharmaceutical treatments on their animals [17]; consumers are concerned with the ethical and sustainable production of food, encouraging the adoption of complementary management strategies by sheep producers rather than relying on anthelmintics alone.

A potential strategy for the long-term management of GIN parasitism is selecting sheep for increased resistance to GIN [18,19,20,21]. There is evidence that some sheep are genetically adapted to survive better and maintain productivity despite GIN infection (i.e., GIN tolerance) or even resist GIN infection altogether, even when subjected to a strong environmental challenge [22,23,24,25,26]. Tolerance and resistance to GIN infection are developed through the interaction of the innate and adaptive arms of the immune system [23]. One mechanism of the innate immune system used to evade GIN infection is the production of cytokines (e.g., interleukin (IL) *IL-4*, *IL-5*, *IL-25*, and *IL-23*) that induce increased gut motility to expel ingested parasites and increase gastrointestinal mucus production to prevent GINs from attaching to the gastrointestinal lining [5,24]. Additionally, innate immunity to helminth infection in sheep is characterized by the expansion of innate immune cells, including mast cells and eosinophils, which degranulate to release reactive oxygen species, histamine, proteases, and chemokines [24]. The adaptive immune system is the arm of the immune system involved in immunological memory, conferring lasting protection against a recognized pathogen such as GINs. The polarization of the adaptive immune system towards a type-2 immune response is associated with GIN resistance [27]. This involves increasing the presence of T-helper (Th-2) cells that influence the production of mucosal IgA and IgE, which are important for reducing the parasite load [5].

The acute-phase response has been implicated as a significant element of the immune response that affects the level of host resistance to GIN infection [24,28]. The liver is a major target of many acute-phase inflammatory molecules, as it is the organ that provides essential metabolites, such as cholesterol and other steroids, to the host during times of stress [29]. The liver also functions in assisting with the clearance of harmful metabolites and aiding in tissue repair during the acute-phase response [30]. Liver cells are sensitive to cytokine signaling molecules of the acute-phase response and participate in signal transduction, modulating the progression of the immune response to infection [31]. Molecules and cells involved in the acute-phase response may influence the development of the subsequent acquired immune response. The liver also responds to proinflammatory cytokines by changing metabolic activity to better reflect the needs of the host during times of acute and chronic stress [29]. Many studies have explored differences in gene expression in tissues directly affected by GIN parasitism, such as the abomasal lymph nodes, abomasal mucosa, and intestinal lining. The hepatic transcriptome may provide additional information on the immune response to GIN parasitism at a more systemic level. The liver is, therefore, an appropriate target tissue for the study of gene and transcript expression during the ovine immune response to GINs.

Understanding the genetic mechanisms controlling the host immune response to GIN infection can help to better inform breeding decisions and future selection programs [19,32]. Insight into this complex phenotype can be achieved through the use of high-throughput sequencing technologies, such as RNA-Sequencing (RNA-Seq). RNA-Sequencing can be used to characterize the transcriptome of a particular tissue or cell type and identify genes that are differentially expressed (DE) between individuals exhibiting different phenotypes or that are subject to different experimental treatments [33]. For example, performing RNA-Seq analysis on the tissues of sheep that have been identified as resistant or susceptible to GIN infection can provide insight regarding the genes that function in and regulate the host response and subsequently help identify the key biological pathways associated with GIN resistance.

Therefore, the objectives of this study were as follows:

1. Identify hepatic genes differentially expressed between sheep characterized as having a high acute-phase immune response and sheep characterized as having a medium acute-phase immune response that has been naturally infected with GIN, as well as differentially expressed genes between these sheep and GIN-naïve sheep,
2. Identify hepatic genes that are differentially expressed between sheep characterized as having an antibody-mediated bias immune response and sheep characterized as having a cell-mediated bias immune response that has been naturally infected with GIN, as well as differentially expressed genes between these sheep and GIN-naïve sheep,
3. Integrate the results of each experiment to identify potential key regulator genes associated with the host immune response to GIN infection.

## 2. Materials and Methods

### 2.1. Animal Husbandry and Sample Collection

#### 2.1.1. Experiment One

The sheep used in this study were part of a larger study conducted at the Ontario Veterinary College (Guelph, ON, Canada) that evaluated the immune response of lambs to natural infection with GINs [33]. The ethical use of animals was approved for all procedures by the University of Guelph Animal Care Committee (Animal Use Protocol Number 3380).

The animals used in this study were Rideau x Dorset lambs born and housed at the Ontario Sheep Research Centre (Ponsonby, ON, Canada). Animals were considered to be GIN-naïve as the source of the flock was specific-pathogen free. All lambs born between April and November 2015 (*n* = 180) were challenged at 60 days of age with an intravenous infusion of 0.4 g/kg *Escherichia coli* lipopolysaccharide (LPS) (O111:B4 E. coli_L2630_SigmaAldrich) based on the protocol described in 2008 by [34] to stimulate their acute-phase (AP) immune response. High AP-responding (H-AP; *n* = 15) and medium AP-responding (M-AP; *n* = 15) lambs were selected based on their change in serum cortisol concentration 4 h post-infusion with LPS; H-AP lambs had cortisol concentrations greater than one standard deviation (SD) above the population mean (>211.3 nmol/L, cortisol range 242–494 nmol/L), and M-AP lambs had the smallest absolute difference between their peak cortisol concentration and the population mean peak cortisol concentration (117.0 nmol/L, cortisol range 72–152 nmol/L) [34]. The comparison of H-AP lambs and M-AP lambs instead of low AP responders was elected as type I and type II immune responses were more similar between lambs with an extreme cortisol response than between either high or low and medium cortisol response [30]. Animals with deformities that would prevent normal grazing behavior, such as angular limb deformity or jaw misalignment, were excluded from the study [35].

On 28 April 2016, the H-AP (*n* = 15) and M-AP (*n* = 15) lambs (*n* = 30, age range 160–380 days) were turned out on pasture in central Ontario, Canada, with a group of commercial Rideau x Dorset ewes and lambs at a farm with a known history of GIN parasitism. Study lambs were naturally exposed to GINs by grazing with the commercial flock over the course of the grazing season, which ended on 3 November 2016 (189 days on pasture). The average FECs between the H-AP and M-AP groups did not differ at any time point during the grazing season [33]. Individual FECs were zero at the start of the study period. The mean FECs increased from 12 to 74 days on pasture to approximately 60 eggs per gram (epg) and increased again sharply from 75 to 117 days on pasture, at which point they peaked at approximately 880 epg. FECs decreased from this point onward until the end of the experiment, decreasing sharply from 118 days on pasture to 172 days (approximately 40 epg) and remaining consistently low until the end of the experiment [33]. One M-AP animal was removed from the study in August 2016 due to an illness unrelated to GIN infection. Additionally, eight sheep (all medium acute-phase responders, mean peak cortisol = 111.38 +/− 35.6 nmol/L) were kept at the research station and grazed on GIN-free pasture as uninfected controls (U) from April to November 2016. These animals were not infected with GIN parasites but had constant access to pasture; their parasite-free status was confirmed with individual fecal egg counts (FECs) and with the visual inspection of GI tracts for mature and immature worms post-slaughter [28].

Parasite-exposed (*n* = 29) and control (*n* = 8) lambs were euthanized in November 2016 by electrical stunning followed by exsanguination. At the time of slaughter, no animals displayed signs of heavy parasite burdens [33]. The gastrointestinal tract was removed from the carcass and visually inspected for mature and immature parasites. At the same time, approximately 5 g of the right liver lobe was removed with a sterile scalpel, washed in a phosphate-buffer solution (PBS), and stored in liquid nitrogen for transportation. Liver samples were then stored at −80 °C until RNA could be isolated [28].

**Figure 1:**
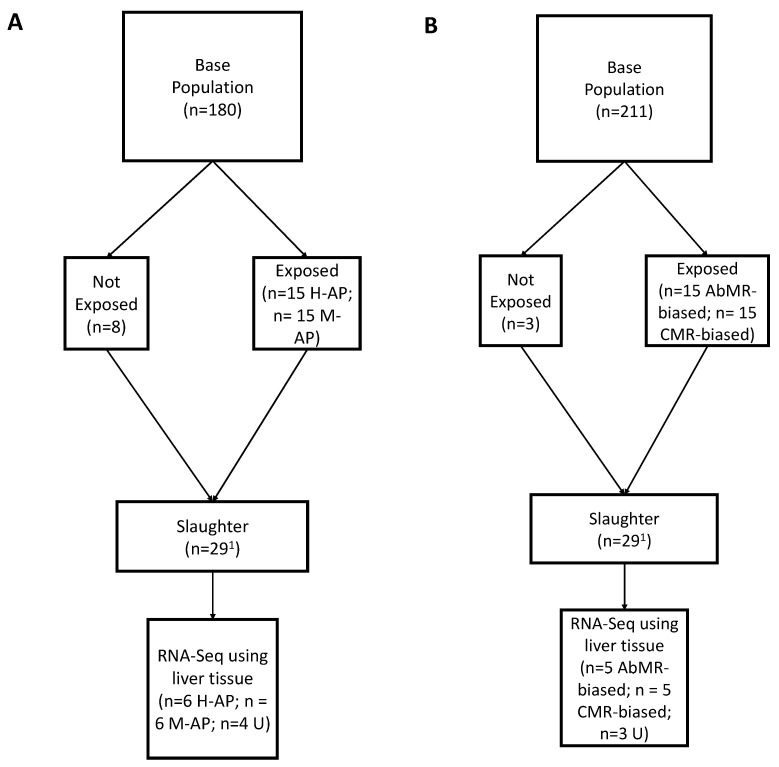
Schematic of experimental protocol for the comparison of hepatic gene expression in sheep naturally exposed to nematodes with varying immune response phenotypes based on (**A**) innate immune responsiveness (experiment 1) and (**B**) adaptive immune responses (experiment 2). ^1^ One animal removed from each experiment due to illness unrelated to parasitemia.

Animals in the H-AP and M-AP groups with the most extreme (highest and lowest) parasite burdens based on a visual inspection of the gastrointestinal tract were selected for RNA-Seq (H-AP = 6, M-AP = 6). Four animals from the U group without any GINs were chosen as the control group [28]. Figure 1A depicts a summary of the protocols used in Experiment 1.

#### 2.1.2. Experiment Two

A similar workflow was followed for experiment two, except that lambs were classified based on their antibody-mediated immune responsiveness (AbMR) and cell-mediated immune responsiveness (CMR) instead of their innate AP-responsiveness.

High AbMR/lowCMR (AbMR-biased) responders and low AbMR/highCMR (CMR-biased) responders were selected from 211 lambs born at the Ontario Sheep Research Centre (Ponsonby, ON, Canada) in 2016. Animals were intramuscularly challenged with 0.5 mg of hen egg white lysozyme (HEWL) + 0.5 mg Quil A to assess AbMR and 0.5 mg of Candida albicans antigen (CAA) + 0.5 mg Quil A intradermally to assess CMR twice, two weeks apart, according to the protocol by Thompson-Crispi & Mallard [36]. The AbMR response was assessed based on the serum IgG response to HEWL 21 days post-challenge, and the CMR was assessed by the magnitude of change in skin thickness 48 h post the intradermal challenge with CAA. Fifteen lambs with skin thickness changes greater than 1 SD above the flock mean and anti-HEWL IgG greater than 1 SD below the population mean were chosen as CMR-biased animals, and 15 lambs with changes in skin thickness greater than 1 SD below the mean and anti-HEWL IgG greater than 1 SD above the population mean were chosen as AbMR-biased animals. As in experiment one, animals with deformities that would prevent normal grazing behavior were not included in the study [33]. The AbMR-biased and CMR-biased animals were turned out on pasture at approximately one year of age to co-graze with the same flock of Rideau x Dorset ewes and lambs that were known to have GIN parasites, as used in experiment one. The study lambs were on pasture from 27 April 2017 to 9 November 2017 (196 days on pasture) and were naturally exposed to GINs during this period. The mean FECs were similar between the high AbMR-biased and high CMR-biased groups across the grazing season [33]. In both groups, mean FECs were between 100 and 225 epg when first sampled at 26 days on pasture (May), peaked at between 300 and 400 epg at 74 days on pasture (July), and decreased to low levels (<50 epg) from 116 days on pasture (August) until the end of the study at 196 days on pasture (November). One AbMR-biased lamb was euthanized prior to the end of the study due to reasons unrelated to parasitic gastroenteritis. The remaining study lambs did not develop clinical signs of illness for the remainder of the study [33]. Additionally, three U animals from the same cohort were kept at the Ontario Sheep Research Centre. These animals had access to pasture over the same time period (27 April 2017 until 9 November 2017) but were not exposed to GINs [33].

All lambs, including the 29 exposed (*n* = 14 AbMR-biased and *n* = 15 CMR-biased animals) and 3 unexposed control sheep, were euthanized by electrical stunning followed by exsanguination at the end of the grazing season. The gastrointestinal tract was immediately removed for analysis of the parasite burden. Also, 5 g of right lobe liver tissue was collected, washed in PBS, and stored in liquid nitrogen for transportation. Liver samples were kept at −80 °C until RNA isolation could be performed [28].

Abomasal immature worm counts were used to identify phenotypically divergent individuals for RNA-Seq as the burden of arrested larval worms best-reflected parasite infection at the time of euthanasia [37]. AbMR-biased (*n* = 5) and CMR-biased (*n* = 5) animals with extreme burdens were chosen for RNA-Seq analysis. A summary of the number of animals selected for each step of experiments one and two can be found in Table 1. Figure 1B depicts a summary of the protocols used in experiment 2.

### 2.2. RNA Extraction

Both experiments one and two used the same RNA extraction protocol, which is detailed along with the RNA-Seq protocol by [28]. Briefly, 30 mg of liver tissue from each of the 5 g samples was ground and homogenized. RNA was extracted from liver cells using Qiagen RNeasy^®^ Mini Prep Kit (Qiagen, Valencia, CA, USA) according to the manufacturer’s instructions. RNA quantity and quality were assessed using a Nanodrop © Spectrophotometer (Thermo Fisher Scientific, Waltham, MA, USA) and an Agilent 2100 Bioanalyzer (Agilent Technologies, Inc., Santa Clara, CA, USA), respectively. All RNA samples used in this study had an RNA integrity value > 8.0, indicating the very good quality of the RNA [38].

### 2.3. Library Construction and RNA-Sequencing

Library preparation with Poly-A selection was completed using the NEBNext^®^ Ultra RNA Library Prep kit for Illumina^®^ (New England Biolabs, Ipswich, MA, USA) according to the instructions from the manufacturer [24]. A Qubit fluorometer (Thermo Fisher Scientific, Waltham, MA, USA) was used to assess library quality and concentration [38], and an Illumina HiSeq 2000 analyzer (Illumina, San Diego, CA, USA) was used for RNA-Seq that resulted in 150 base pair (bp) paired-end reads.

Sequence reads were aligned to the annotated ovine reference genome Oar_rambouillet_v1.0 (Ensembl release 103) using CLC genomics workbench software (CLC Bio, Aarhus, Denmark). Quality control, including the GC content, ambiguous base content, Phred score, base coverage, nucleotide 35 contributions, and overrepresented sequence parameters, was carried out according to the protocol outlined by [39]. One sample (H-AP individual from experiment one) did not pass the quality control due to a large number of uncounted fragments (12%); the rest of the samples passed the quality control analysis based on the following criteria: homogenous read length (150 bp), 100% coverage of all bases, equal representation (25% each) of every nucleotide A, T, G, and C, and less than 0.1% of overrepresented sequences [39]. Transcript levels were transformed to reads per kilobase per million mapped reads (RPKMs) and normalized using a log2 transformation [40]. This allowed for an unbiased gene expression comparison between the experimental groups in studies one and two [41].

### 2.4. Differential Gene Expression Analysis

#### 2.4.1. Experiment One

Differential gene expression analysis was performed between uninfected control (*n* = 4) and M-AP (*n* = 6) sheep, between uninfected control (*n* = 4) and H-AP (*n* = 5) sheep, and between M-AP (*n* = 6) and H-AP (*n* = 5) sheep. Differential gene expression analysis was performed using the “Empirical analysis of Differential Gene Expression” tool in CLC Bio. Genes were considered DE when they had a false discovery rate (FDR) ≤ 0.05 and a fold change |FC| > 2. Associated gene name annotation on each list was performed using the Ensembl biomart tool (http://useast.ensembl.org/index.html, accessed on 1 January2023).

Additionally, genes were indexed into categories based on their expression level within each experimental group and based on their distribution for the group mean RPKM: high expression (>1 SD above the mean), medium expression (within 1 SD of the mean), and low expression (>1 SD below the mean).

#### 2.4.2. Experiment Two

The same workflow was followed for the DE expression analysis in study two, except for the number of animals compared in each group. In study two, differential gene expression analysis was performed between uninfected control (*n* = 3) and CMR-biased sheep (*n* = 5), between uninfected control (*n* = 5) and AbMR-biased (*n* = 5) sheep, and between CMR-biased (*n* = 6) and AbMR-biased (*n* = 5) sheep.

### 2.5. In Silico Functional Analysis

For both studies, functional analysis was performed for each comparison, including gene ontology (GO) enrichment analysis, metabolic pathway analysis, and gene network analyses, which were performed using STRING [42] (https://string-db.org, accessed on 15 January 2023) and *Ovis aries* as the organism. Each unique gene list was uploaded to STRING using the “Multiple proteins by names/identifiers” tab. A protein–protein interaction (PPI) network was created using the default parameters, and upon completion, the network was filtered to include genes with a high confidence PPI score (0.7). Gene ontology analysis, including the three main GO categories (biological function (BP), molecular function (MF), and cellular component (CC)), were retrieved [43] and were used in addition to metabolic pathways enriched within the PPI network.

To identify potential key-regulator genes, transcriptomic and functional data were integrated using a Venn diagram and the following criteria: (1) genes that were DE and had an RPKM value > 1 SD above the group mean, and (2) genes that were common to all comparisons.

## 3. Results

### 3.1. Summary Statistics of the RNA-Sequencing Analysis

#### 3.1.1. Experiment One

Approximately 1 billion paired-end reads were generated from the liver tissue samples (*n* = 13). Each sample yielded an average of ~77 million reads, approximately 95% of which were mapped in pairs. The RNA-Seq yielded an average of ~27 million uniquely mapped fragments, of which approximately 77% were aligned with the ovine reference genome (release 103, 25,145 genes; Table 2).

#### 3.1.2. Experiment Two

The summary of statistics for the RNA-Seq analysis was similar between the two studies. In study two, approximately 1 billion paired-end reads were generated from the liver tissue samples (*n* = 13). Each sample yielded an average of ~77 million reads, approximately 95% of which were mapped in pairs. The RNA-Seq yielded an average of ~34 million uniquely mapped fragments, of which approximately 86% were aligned to the ovine reference genome (release 103; 25,145 genes; Table 2).

### 3.2. Differentially Expressed Genes between Experimental Groups

#### 3.2.1. Experiment One

The total number of DE genes expressed in the samples from study one was 0, 136, and 167, between the H-AP and M-AP animals, the H-AP and U animals, and the M-AP and U animals, respectively. Because there were no DE genes identified between the H-AP and M-AP animals, this comparison was excluded from the rest of the analyses. For the comparison between the H-AP and U animals, the most upregulated gene based on FC was *ENSOARG00020004512* (FC = 75.25, FDR < 0.05). The most downregulated gene for this comparison was *ENSOARG00020009021* (FC = −44.59, FDR < 0.05). For the comparison between the M-AP and U animals, the most upregulated gene based on FC was fibroblast growth factor 21 (FGF21; FC = 24.71, FDR < 0.05). The most downregulated gene for this comparison was *ENSOARG00020009021* (FC = −96.39, FDR < 0.05). A complete summary of the DE genes for both comparisons can be found in Supplementary Tables S1 and S2, respectively.

#### 3.2.2. Experiment Two

The total number of DE genes expressed in the samples from study two were 0, 53, and 57 between the AbMR- and CMR-biased animals, the AbMR-biased U animals, and the CMR-biased and U animals, respectively. Again, there were no DE genes identified for the comparison between the two groups of GIN-exposed sheep; thus, this comparison was excluded from the rest of the analyses. For the comparison between the AbMR-biased and U animals and the comparison between the CMR-biased and U animals, the most upregulated gene based on FC was *ENSOARG00020004512* (FC = 51.72, FDR < 0.05; FC = 75.25, FDR < 0.05, respectively). The most downregulated gene for both of these comparisons was *ENSOARG00020009021* (FC = −47.20, FDR < 0.05 and −44.59, FDR < 0.05). Supplementary Tables S3 and S4 contain a complete summary of the DE genes for both comparisons in experiment two (i.e., DE genes between AbMR-biased and uninfected control animals and DE genes between CMR-biased and uninfected control animals, respectively).

### 3.3. Gene Network Analysis

#### 3.3.1. Experiment One

**Figure 2:**
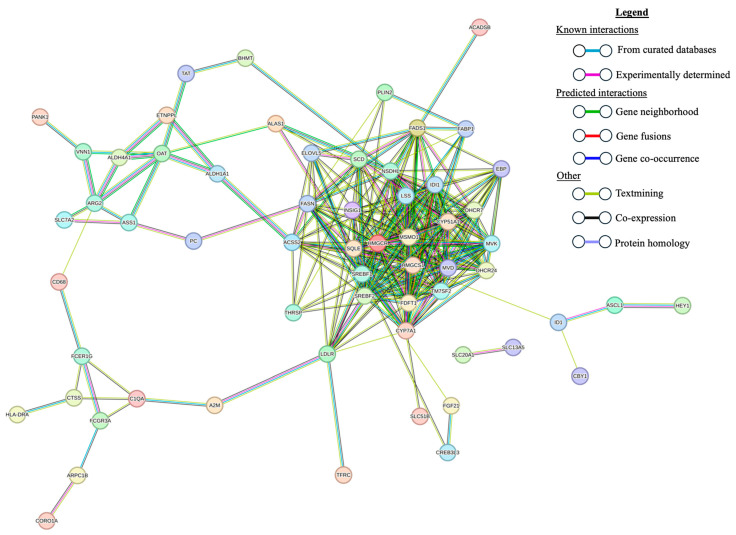
Protein–protein interaction network constructed with STRING using the 136 differentially expressed genes in liver tissue from high-acute-phase-responsive sheep naturally exposed to nematode parasites and unexposed controls. Disconnected nodes are not shown.

**Figure 3:**
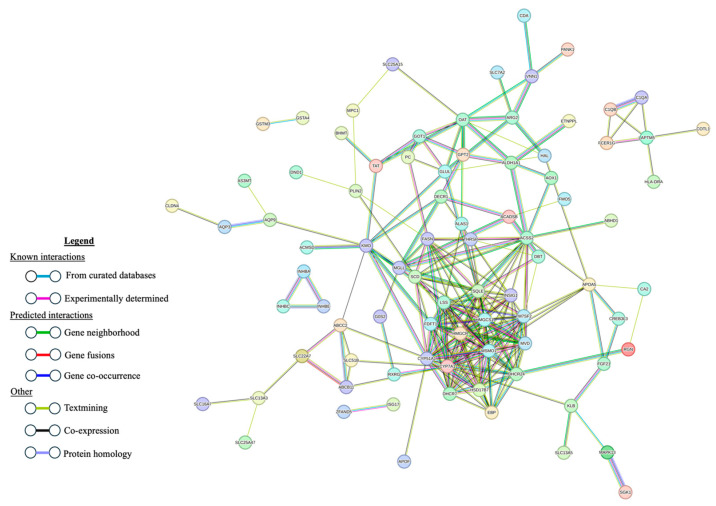
Protein–protein interaction network constructed with STRING using the 167 differentially expressed genes in liver tissue from medium-acute-phase-responsive sheep naturally exposed to nematode parasites and unexposed controls. Disconnected nodes are not shown.

The STRING PPI network generated from the list of 136 DE genes between the H-AP and U animals had 86 nodes (genes) and 250 edges (interactions; Figure 2). Eleven of these eighty-nine genes were also DE in the hepatic transcriptomes of H-AP and U animals. For the comparison of the M-AP and U animals, the PPI generated by STRING from the 167 DE genes contained 106 nodes and 235 edges (Figure 3). Of these, 11 genes were also DE between H-AP and U sheep.

#### 3.3.2. Experiment Two

**Figure 4:**
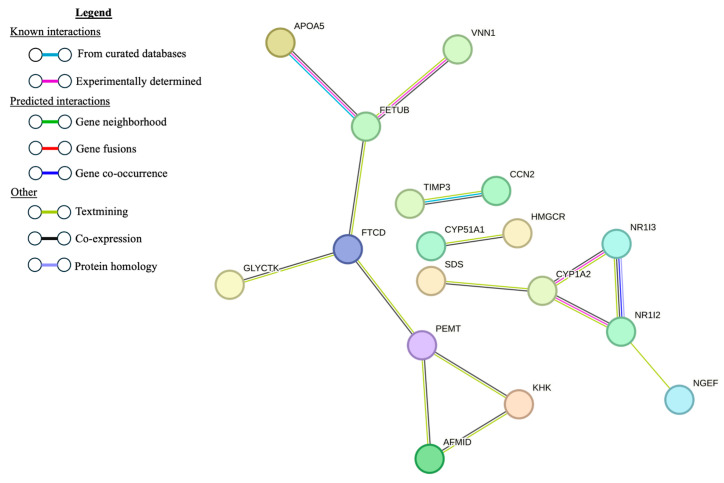
Protein–protein interaction network constructed with STRING using the 53 differentially expressed genes in liver tissue from antibody-mediated immune-response-biased sheep naturally exposed to nematodes and unexposed controls. Disconnected nodes are not shown.

**Figure 5:**
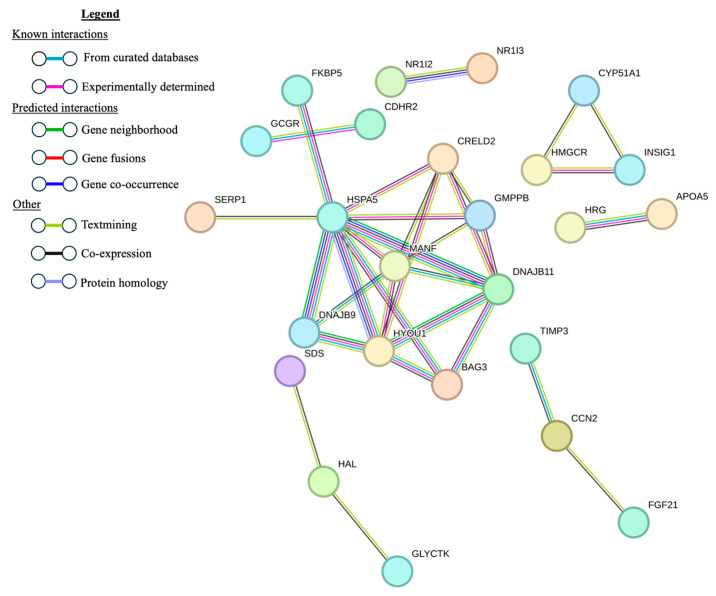
Protein–protein interaction network constructed with STRING using the 57 differentially expressed genes in liver tissue from cell-mediated immune-response-biased sheep naturally exposed to nematodes and unexposed controls. Disconnected nodes are not shown.

Using the list of 53 DE genes between the AbMR-biased and U animals, the PPI generated by STRING had 38 nodes and 7 edges (Figure 4). Six of the genes identified by STRING were highly expressed DE hepatic genes. The PPI generated by STRING from the list of 57 DE genes between the CMR-biased and U animals had 45 nodes (genes) and 32 edges (Figure 5). Of these 46 genes, 9 were highly expressed DE genes in the liver.

### 3.4. Gene Ontology Enrichment and Metabolic Pathway Analysis

#### 3.4.1. Experiment One

Gene ontology enrichment analyses were performed using the entire list of DE genes in each comparison. For the comparison between the H-AP and M-AP animals, 47, 10, and 5 significantly enriched GO terms were retrieved from the BP, MF, and CC categories, respectively. Overall, the most significant BP terms based on strength (i.e., Log10 (n_observed_/n_expected_) [42] were the Isopentenyl diphosphate biosynthetic process, mevalonate pathway, and cholesterol biosynthetic process. There were 10 enriched KEGG terms associated with the PPI network. The three terms with the highest strength were steroid biosynthesis, terpenoid backbone biosynthesis, and the biosynthesis of unsaturated fatty acids.

For the comparison between the M-AP animals and U animals, there were 56 enriched BPs, 22 enriched MFs, and 1 enriched CC identified. The antibiotic metabolic process, cholesterol biosynthetic process, and sterol biosynthetic process were the BP GO terms that were most significant for the comparison between M-AP and uninfected control animals. The genes in the PPI network were associated with 17 metabolic pathways, of which the four most significant were steroid biosynthesis, phenylalanine, tyrosine and tryptophan biosynthesis, and arginine biosynthesis based on their strength. Enriched metabolic pathways for both comparisons in experiment one are presented in Table 3.

#### 3.4.2. Experiment Two

The same parameters and workflow were used for study two as for study one. For the comparison between AbMR- and CMR-biased animals, only three enriched BPs were identified. They were the cholesterol metabolic process, small molecule metabolic process, and alcohol metabolic process. There were no enriched metabolic pathways identified in this PPI network.

For the comparison between the CMR-biased and uninfected control animals, there were 13 enriched BPs and one enriched MF. The most enriched BPs were the negative regulation of the ire1-mediated unfolded protein response, the cholesterol biosynthetic process, and the response to ischemia. Neither comparison yielded significant CCs (FDR > 0.05). There was one enriched metabolic pathway associated with this PPI glycine, serine, and threonine metabolism.

### 3.5. Potential Key Regulator Genes

Potential key regulator genes for each study were identified from the lists of DE genes and were defined as those genes that had greater expression levels based on RPKM (i.e., >1 SD above the mean), present in the PPI network, and which were present in all comparisons in that experiment (i.e., the comparison between the two exposed phenotypes and between each exposed phenotype and the uninfected control animals). In study one, there were 6 genes that fitted these criteria: 5′ aminolevulinate synthase 1 (*ALAS1*), aldehyde dehydrogenase 1 family member A1 (*ALDH1A1*), betaine homocysteine (*BHMT*), cyclic adenosine monophosphate response element binding protein-like 3 (*CREB3L3*), solute carrier family 25 member 47 (*SLC25A47*), and tyrosine aminotransferase (*TAT*). There were no genes that met these criteria in study two.

## 4. Discussion

Infection with GIN is a problem for sheep production worldwide as it decreases the production efficiency of the operation and compromises the welfare of infected animals. Complementary methods of managing GIN parasitism, aside from relying on the use of anthelmintics, are needed in order to maintain the sustainability and viability of sheep production. A potential strategy is to genetically select sheep that have a naturally enhanced immune response to these parasites and are able to resist infection. This study sought to characterize the innate immune response of GINs using sheep phenotyped for their innate acute-phase response level and the adaptive antibody-mediated and cell-mediated immune response to GINs via a transcriptomics approach. The results described herein have elucidated and improved our understanding of some of the genetic architecture and biological mechanisms controlling these traits.

### 4.1. Innate Responses

Unexpectedly, there were no genes that were DE between the H- and M-AP animals. It is likely that there were differences in the genetic regulation of the host immune response to the parasite challenge, but these were not captured in the dataset. Indeed, the most active immune organ involved in the innate immune response to parasites is the abomasal mucosa, which is a tissue directly affected by infection with GINs [44]. However, it was hypothesized that there would still be some differential gene expression at the hepatic level, as all the blood and metabolites that pass through the abomasum subsequently pass through the liver via the portal vein and could potentially induce transcriptional differences. The liver also actively functions in the acute-phase response of the innate immune system, as studied by [45]. It is responsible for the production of acute-phase proteins that are associated with immune response, such as cytokines and chemokines. Similarly, study [28] found that there was no differential gene expression in liver tissue samples of sheep that were exposed to GIN but which had differing levels (high versus low) levels of infection. Thus, the current data suggest that during the chronic or long-term infection period, gene expression in the liver is likely less important than in the short-term/acute-phase of infection.

When comparing the exposed animals with uninfected controls, 136 and 167 genes were DE between the H-AP animals and the U animals and the M-AP animals and the U animals, respectively. The most upregulated gene based on FC in the comparison between H-AP and control animals was *ENSOARG00020004512*. This is a novel gene in the ovine reference genome, and its function has not been annotated. However, the orthologue found in mice (*Mus musculus*), ribosomal protein 25 (*Rpl23*), is associated with cell proliferation, transcription, response to stimulus, and signaling (Mouse Genome Informatics, https://www.informatics.jax.org/, accessed on 15 December 2022). It is a negative regulator of cellular apoptosis and is related to oncogenesis in humans [46]. Another interesting upregulated (FC = 5.56) but lowly expressed (mean RPKM = 6.76) gene in H-AP individuals is the C-X-C motif chemokine ligand 9 (*CXCL9*) gene. This gene is likely involved in the innate response to GIN infection, as it was found to be upregulated in GIN-resistant sheep in other studies [47,48]. In the comparison between the M-AP sheep and the uninfected control sheep, the most upregulated gene was *ENSOARG00020009021* based on FC. The function of this gene has not been annotated in sheep, but it has orthologues in mice and humans (*ENSMUSG00000029119* and *ENSG00000013288*, respectively) associated with combined immunodeficiency and dysregulation [49]. Another interesting, upregulated gene in the M-AP group is the thymine β 10 (*TMSB10*) gene (FC = 2.50, mean RPKM = 307.88). This chemotaxic gene has been associated with immune cell trafficking in swine challenged with LPS [50] and is downregulated in human cells infected with *Mycobacterium bovis* [51]. It is possible that the dysregulation of *TMSB10* (under- or over-production) can lead to the impairment of immune function. The role of this gene in the innate immune response to GINs, therefore, merits further investigation.

In terms of GO, many of the BPs associated with H-AP animals compared with uninfected control animals related to biosynthetic processes. The isopentenyl diphosphate biosynthetic process, cholesterol biosynthetic process, isoprenoid biosynthetic process, organic hydroxy compound biosynthetic process, lipid biosynthetic process, fatty acid biosynthetic process, and regulation of lipid biosynthetic process were all significantly enriched in BPs (FDR < 0.05). This is to be expected, as the liver is primarily an organ involved in metabolism, nutrient storage, and detoxification [45]. Another enriched BP in H-AP sheep was response to the lipoprotein particle, indicating that these sheep likely had a strong innate immune response [52]. An important MF term identified in the H-AP sheep was oxidoreductase activity, which is related to the response to reactive oxygen species [53]. The expression of genes involved in oxidoreductase activities in a response to LPS has been found to induce the expression of both pro- and anti-inflammatory cytokines. Lastly, the four enriched CC terms associated with the comparison of the H-AP animals to the U animals were the endoplasmic reticulum (ER) membrane, the endomembrane system integral component of the endoplasmic reticulum membrane, organelle membrane, and SREBP-SCAP-Insig complex. The enrichment of these terms indicated that the DE genes identified in this comparison are likely to have an effect on the regulation of lipoprotein synthesis, which has increasingly been recognized as having a role in immune function and modulation [54,55]. Similarly, many of the enriched metabolic pathways associated with the H-AP versus the uninfected control animal comparison are related to biosynthetic processes (steroid biosynthesis, terpenoid backbone biosynthesis, and biosynthesis of unsaturated fatty acids) and lipid metabolism (fatty acid metabolism). immune-related metabolic pathways identified in this comparison included phagosome and tuberculosis. Phagosome enrichment may be related to the upregulation of macrophage expression in H-AP animals, which act as regulatory cells in the immune response, repair damaged tissue, and prevent larvae from colonizing the abomasum in GIN-challenged sheep [5,56] through the processing and presentation of phagocytized antigens to T-cells [57].

For the comparison between the M-AP animals and the U animals, many enriched BPs were related to amino acid catabolism, including the glutamine family amino acid catabolic process, aromatic amino acid family catabolic process, α-amino acid catabolic process, and cellular amino acid catabolic process. This could indicate that lambs were undergoing tissue remodeling (i.e., protein catabolism) to produce the necessary immune proteins for an effective immune response to GIN infection [58,59]. Again, oxidoreductase activity was enriched in this comparison, highlighting the importance of this biological process in the host immune response to GINs. A large number of enriched MFs were related to molecule binding, such as vitamin binding, pyridoxal phosphate binding, small molecule binding, amide binding, anion binding, and FAD binding. These likely reflect attempts by the host to modulate the activity of certain metabolic and immunological molecules [29]. The sole-enriched cellular component in this comparison was the endoplasmic reticulum membrane. The ER is an organelle known to function in the synthesis of secretory and membrane proteins [60], and changes in its morphology and function are thought to play a role in the immune response to pathogens in humans. Similar to the enriched MFs, many of the enriched metabolic pathways were related to amino acid metabolism, including phenylalanine, tyrosine and tryptophan biosynthesis, arginine biosynthesis, tyrosine metabolism, alanine, aspartate and glutamate metabolism, tryptophan metabolism, arginine and proline metabolism, valine, leucine and isoleucine degradation, cysteine and methionine metabolism, and the biosynthesis of amino acids. This further supports the notion that changes in protein metabolism occur in M-AP animals.

### 4.2. Adaptive Responses

The adaptive immune response to infection is not as quick to develop as the innate immune response, but it is associated with longer-lasting protection, antigen-specific defense, and immunological memory regarding subsequent exposures to pathogens [24]. In general, an enhanced AbMR response is associated with immunity to GINs while CMR bias is associated with susceptibility [5,61], although the interplay between the two arms of the adaptive immune system is not perfectly understood, and cytokines associated with one response may influence the other [61]. Additionally, many cytokines and chemokines associated with the innate response also act as signaling molecules for the adaptive response.

Overall, there was reduced differential gene expression observed when comparing animals phenotyped for adaptive responses to those with known innate responses. There were no DE genes identified between the AbMR- and the CMR-biased animals; thus, this comparison was excluded from further analyses. This may be because the liver is not particularly active in the development and modulation of the adaptive immune response to GIN infection in sheep. The most immunologically active tissues would likely be lymph-specific tissues, such as the abomasal lymph nodes [62]. However, as identified by [45] the liver does play an immunomodulatory role, as there are lymphocyte populations within this tissue. This is likely why there was some differential gene expression between the AbMR-biased and U animals (53 DE genes) and the CMR-biased and control animals (57 DE genes). Among the 53 DE genes between the AbMR-biased and U animals and the 57 DE genes between the CMR-biased and control animals, the most upregulated gene was *ENSOARG00020004512* in both cases (FC = 51.72 and 75.25, respectively. This is the same gene that was upregulated when comparing H-AP and U animals in experiment one. For both comparisons (AbMR-biased animals versus U animals and CMR-biased animals versus U animals) in experiment two, the most downregulated gene was *ENSOARG00020009021*, which was also the most downregulated gene when comparing H-AP and M-AP animals to U animals in experiment one. These results highlight the importance of these two genes in the ovine response to helminth infection. Interestingly, the majority of the highly up- and down-regulated genes were not those that were highly expressed in liver tissue (i.e., although there were differences in expression levels between the groups, overall, their expression level was within 1 SD of the mean RPKM of that group). This reflects the fact that the liver may not be a major organ involved in the adaptive immune response to helminths and that although there are physiological differences between the groups, they are not captured by this dataset of transcribed hepatic genes. It is possible that there was more differential gene expression in the liver earlier on in the infection period, but by the end of the grazing season, these differences had subsided [28]. Thus, the differences in expression may provide more information regarding the resolution phase of GIN infection, as opposed to highly active immune responses during peak infection. The timing of slaughter in November, rather than closer to the peak of the grazing season and the subsequent parasite challenge, was a decision based on the experimental design of the large project conducted by [33]. It is possible that some key differences in differential gene expression were already resolved at this stage and, thus, were not captured in the present dataset, which presents an opportunity for future research.

As there was a reduced number of DE genes identified in experiment two, there was also a reduced number of enriched GO terms and metabolic pathways. For the comparison involving the AbMR-biased and uninfected control animals, the following 3 GO terms were identified: the cholesterol metabolic process, small molecule metabolic process, and alcohol metabolic process. For CMR-biased sheep compared with uninfected control sheep, 3 of the 13 enriched GO terms related to protein unfolding are as follows: the negative regulation of ire1-mediated unfolded protein response, response to the unfolded protein, and cellular response to the unfolded protein. Protein folding and unfolding are controlled by the ER, which was also identified as an important mediator in experiment one. Homeostatic changes can cause an accumulation of unfolded proteins in the ER and cause the activation of the unfolded protein response [63]. The Ire-1-mediated unfolded protein response is thought to function by binding specific proteins, including MHC, which would influence the host immune response [63]. Another interesting, enriched BP is the response to ischemia. It has been found that T-cells actively participate in exacerbating ischemic injuries, including in the gastrointestinal tract and liver [64]. Specifically, abnormal Th-17 cells associated with autoimmune disease produced increased amounts of *IL-17*, leading to the induction of pro-inflammatory cytokines and neutrophil recruitment. This further demonstrates the negative impact of a CMR-biased immune response on host resistance to GIN infection in sheep. No enriched MFs were identified in this comparison, and the endoplasmic reticulum lumen was the only enriched CC. One enriched metabolic pathway was identified, glycine, serine, and threonine metabolism, again indicating the importance of amino acid synthesis to the host immune response to GINs.

### 4.3. Potential Key Regulator Genes

Although no genes that met all the outlined criteria were found to be in common among the two experiments, there were six genes identified as potential key regulators in experiment one. These were ALAS1, ALDH1A1, BHMT, CREB3L3, SLC25A47, and TAT. ALAS1, ALDH1A1, SLC25A47, and TAT were downregulated in H-AP animals compared U animals, while only ALAS1, ALDH1A1, and TAT were downregulated in the M-AP sheep compared to the U animals. BHMT, CREB3L3, and SLC25A47 were all upregulated in M-AP sheep compared to U animals. 

Of this list of potential key regulator genes, the most promising functional candidates appeared to be *ALAS1* and *TAT* due to their known involvement in GIN resistance. The *ALAS1* gene has been previously identified as a potential candidate gene associated with indicator traits of GIN resistance in sheep [65]. Study [65] performed a genome-wide association study using the Brazilian Santa Inês breed of sheep using indicator traits, including FAMACHA, hematocrit, white blood cell count, red blood cell count, hemoglobin, platelets, and GIN egg counts per gram of feces. The *ALAS1* gene was found to be involved in the significantly enriched metabolic pathway of glycine, serine, and threonine metabolism, which is associated with immune cell functions, such as inflammation, chemokine signaling, and T-cell activation [65]. The *ALAS1* gene is also associated with heme biosynthesis and can act as a regulatory molecule for hematopoietic and other cells in the liver and associated tissues [66]. Additionally, in an experiment looking at the DE gene expression between GIN-resistant and GIN-susceptible breeds of sheep, *TAT* was DE in both the infected and the uninfected animals of both breeds [67]. Overall, there was higher *TAT* expression in individuals from the GIN-resistant hair breed. TAT is a transcription factor associated with tyrosine catabolism, whose activity is mediated by the presence of glucocorticoids [68].

Other genes that have been identified as potential key regulator genes but which have not yet been associated with the host immune response to GIN infection in sheep are *ALDH1A1, CREBL3*, and *BHMT. ALDH1A1* is a molecule that functions in the RXR/RAR group of ligand-dependent transcription factors [69]. This gene was found to have a major effect on cellular defense against oxidative damage in mouse livers [70]. Additionally, in cells that had *ALDH1A1* inhibited, there was an increase in unfolded protein aggregates. Study [71] found that *BHMT* was associated with fatty acid composition in lambs. This gene is involved in the tissue remodeling of adipose tissue by controlling the movement of lipids from the liver to adipose tissue. The *CREB3L3* gene is also associated with lipid metabolism and is found in the ER in both the liver and small intestine in humans [72].

Although it met the filtering criteria for potential key regulatory genes in the present study, *SLC25A47* likely should not be prioritized as a key regulatory gene for future studies. The SLC family of genes is a mitochondrial transporter family made up of 24 subfamilies and is widespread among eukaryotes [73]. These proteins can transport a variety of solutes with families categorized by substrate specificity. The function of *SLC25A47* is unknown [74], but it likely functions in energy metabolism.

It is interesting that there were no identifiable differences in hepatic gene expression between the groups of GIN-exposed sheep (i.e., between the H-AP and M-AP animals in experiment 1 or between the AbMR-biased and CMR-biased animals in experiment 2). Instead, the results of these experiments point to differences in lipid metabolism between GIN-exposed and GIN-naïve animals. Infection with helminths is known to cause a variety of physiological, metabolic, and immunological changes in the host that allow energy to be readily available to respond to the infection [75,76]. Particularly relevant for Experiment 1, it is known that glucocorticoid levels during helminth infection, including cortisol, can affect host adipose tissue composition and gluconeogenesis [77]. Additionally, Th2 cells are sensitive to glucose and lipid metabolism, as they express genes related to these metabolic processes, as discussed by [76]. Fatty acid synthesis can contribute to the activation and differentiation of T helper cells through the production of increased reactive oxygen species. In humans, white adipose tissue is a site of M2 macrophage activity, which is an active player in the immune response to helminths [78]. Additionally, human adipocytes have been reported to produce *IL-33*, which is an alarmin associated with Th2 polarization during helminth infection. The *IL-33* can also influence the switch of white adipose tissue to beige adipose tissue during helminth, which is a physiological change usually associated with cold stress [76]. Study [28] found that GIN-exposed sheep experienced an increased expression of hepatic genes associated with immune processes and a decrease in genes associated with metabolic processes when compared to unexposed sheep. This study also found marked differences in the expression of genes related to lipid metabolism between GIN-exposed and unexposed animals. In a study conducted on the pathogenicity of dust mites in human airways, [79] found that metabolic pathways associated with lipid metabolism were highly enriched in Th2 cells. A polarized Th2 response is associated with protective immunity against GINs; thus, although not explicitly outlined by our dataset, the results of our study may indicate that lipid metabolism is important to the polarization of the immune response of a type-2 response to garner protective immunity in grazing sheep. In a study of fur seal (Arctocephalus australis), pups infected with hookworms, [77], found that infected pups had slightly lower cholesterol levels than pups that had been treated, potentially as a result of metabolic changes within the host. These metabolic changes are important because they can result in more energy being available to the host in order to mount an effective immune response. Different infectious agents can cause different metabolic changes within the host, so as to monitor the different ways that the phenotype of infected animals may be useful for producers to make informed management decisions within their flocks. More research into host–parasite interactions could help with the identification of useful phenotypes to monitor, such as body weight, the body condition score, and FAMACHA © score for monitoring and eventual selection of potentially resistant animals.

It is possible that differences in gene expression occur within other tissues which were not captured by this dataset. Though the liver receives significant blood flow from the gastrointestinal tract [45] and, therefore, experiences transcriptomic modifications during GIN infection, it is not the primary organ affected by GIN infection in ruminants. However, systemic changes in host metabolism and immune status were expected to be observed. The gastrointestinal mucosa and abomasal lymph nodes may provide information on the specific immune molecules associated with GIN infection, especially as it relates to the development of protective antibodies that protect against subsequent GIN infections [80]. Nonetheless, systemic differences between host metabolism and immune function between high, low, and medium immune responders were expected to be captured in this dataset; however, this was not observed.

## 5. Conclusions

In the first RNA_Seq experiment, a total of 0, 136, and 167 genes were DE between the high and medium AP animals, high AP and uninfected control animals, and medium AP and control animals, respectively, in the first experiment. In the second experiment, 0, 53, and 57 genes were DE between the AbMR- and CMR-biased animals, the AbMR-biased and uninfected control animals, and the CMR-biased and control animals, respectively. Functional analyses were conducted, and several GO terms and metabolic pathways were identified and associated with each gene list. Differences in the expression of genes related to lipid metabolism between GIN-infected and uninfected animals were observed. Six potential candidate genes that modulated the innate immune response to the helminth infection were identified, including *ALAS1, ALDH1A1, BHMT, CREB3L3, SLC25A47*, and *TAT*. Additional research should be conducted to confirm the function of each of these genes in an ovine model. Further investigation could identify SNPs and structural variants associated with these genes for potential use in selection programs for increased resistance to GIN.

## Figures and Tables

**Table 1:** Summary of the number of animals selected for comparison of hepatic gene expression in sheep naturally exposed to gastrointestinal nematode parasites with differing innate and adaptive immune response phenotypes.

	Number of Animals	
	Experiment One (Innate Responses)	Experiment Two (Adaptive Responses)
**Source population**	180	211
Animals selected	38	33
Exposed	30	30
Phenotype 1 ^1^	15	15
Phenotype 2 ^2^	15	15
Unexposed control	8	3
**Animals chosen for RNA-Seq**	16	13
Exposed	9	10
Phenotype 1	6	5
Phenotype 2	6	5
Unexposed control	4	3
**Animals used in analysis**	15	13
Exposed	9	10
Phenotype 1	5	5
Phenotype 2	6	5
Unexposed control	4	3

^1^ High acute-phase responsiveness (experiment one) or antibody-mediated response bias (experiment two). ^2^ Medium acute-phase responsiveness (experiment one) or cell-mediated response bias (experiment two).

**Table 2:** Summary statistics of RNA-Sequencing conducted on liver samples obtained from sheep with different innate and adaptive immune response phenotypes following natural exposure to gastrointestinal nematode parasites.

Experiment	Sample ID	Total Number of Reads	% of Total Reads Mapped in Pairs	% of Total Unique Mapped Fragments
1	Unexposed control_206	75,857,532	95.41	85.54
	Unexposed control_243	84,785,824	95.76	86.76
	Unexposed control_262	67,874,160	94.93	87.19
	Unexposed control_273	79,928,750	94.90	84.91
	High AP_1031	72,250,158	96.11	87.05
	High AP_1059	73,792,178	95.36	86.00
	High AP_1117	72,884,046	95.66	87.35
	High AP_1186	71,614,630	93.72	84.81
	High AP_1246	68,912,606	94.13	84.88
	Medium AP_1010	88,738,630	94.70	86.20
	Medium AP_1017	77,347,630	94.59	85.60
	Medium AP_1113	83,933,474	94.15	83.13
	Medium AP_1131	61,255,546	94.15	83.13
	Medium AP_1205	104,382,392	94.69	84.72
	Medium AP_1234	85,849,686	96.41	88.22
	**Averages**	77,960,483	95.00	86.00
	High AP	71,890,724	95.00	86.00
	Medium AP	83,584,560	95.00	85.00
	Unexposed control	77,111,567	95.00	86.00
2	Unexposed control_D83	86,176,142	94.76	76.24
	Unexposed control_D113	68,427,336	95.69	81.00
	Unexposed control_D105	68,486,186	94.85	73.06
	AbMR-biased_1290	86,409,894	95.51	82.82
	AbMR-biased_2919	67,867,826	94.49	79.72
	AbMR-biased_2957	73,484,880	94.94	76.00
	AbMR-biased_2972	84,806,558	95.47	78.71
	AbMR-biased_3006	84,747,062	94.96	80.16
	CMR-biased_3014	77,688,428	93.93	71.93
	CMR-biased_3026	66,233,564	93.96	76.06
	CMR-biased_3033	67,802,374	94.08	80.25
	CMR-biased_3043	79,363,440	94.43	78.92
	CMR-biased_3060	95,225,922	93.73	71.03
	**Averages**	77,439,970	95.00	77.00
	AbMR-biased group	79,463,244	95.00	79.00
	CMR-biased group	77,262,746	94.00	76.00
	Unexposed control	74,363,221	95.00	77.00

AP = acute-phase response (innate immunity). AbMR = antibody-mediated response, and CMR = cell-mediated response (adaptive immunity).

**Table 3:** Significant metabolic pathways (false discovery rate < 0.05) associated with differentially expressed genes in the liver tissues of sheep with varying innate immune response phenotypes naturally exposed to nematode parasites.

Comparison	Pathway Name	FDR	DE Genes	Total Genes in Pathway
High AP versus unexposed control	Steroid biosynthesis	4.24 × 10^−13^	9	18
	Metabolic pathways	7.48 × 10^−13^	30	1263
	Terpenoid backbone biosynthesis	2.80 × 10^−4^	4	19
	PPAR signaling pathway	1.20 × 10^−3^	5	68
	Bile secretion	1.30 × 10^−3^	5	73
	Tuberculosis	2.30 × 10^−3^	6	143
	Fatty acid metabolism	3.80 × 10^−3^	4	50
	AMPK signaling pathway	3.80 × 10^−3^	5	101
	Biosynthesis of unsaturated fatty acids	5.80 × 10^−3^	3	22
	Phagosome	8.80 × 10^−3^	5	129
Medium AP versus unexposed control	Metabolic pathways	7.32 × 10^−17^	38	1263
	Steroid biosynthesis	1.28 × 10^−12^	9	18
	Bile secretion	9.72 × 10^−7^	8	73
	Arginine biosynthesis	2.00 × 10^−4^	4	15
	PPAR signaling pathway	2.50 × 10^−3^	5	68
	Terpenoid backbone biosynthesis	9.50 × 10^−3^	3	19
	Drug metabolism—cytochrome P450	9.50 × 10^−3^	4	50
	Tyrosine metabolism	1.83 × 10^−2^	3	27
	Biosynthesis of amino acids	1.83 × 10^−2^	4	65
	Phenylalanine, tyrosine and tryptophan biosynthesis	2.38 × 10^−2^	2	6
	Alanine, aspartate and glutamate metabolism	2.72 × 10^−2^	3	34
	Signaling pathways regulating pluripotency of stem cells	3.74 × 10^−2^	4	89
	Tryptophan metabolism	3.82 × 10^−2^	3	41
	Valine, leucine and isoleucine degradation	4.04 × 10^−2^	3	44
	Arginine and proline metabolism	4.04 × 10^−2^	3	43
	Cysteine and methionine metabolism	4.24 × 10^−2^	3	46
	Carbon metabolism	4.42 × 10^−2^	4	103

## Data Availability

The datasets generated and/or analyzed during the current study are not publicly available due to the agreements signed with the institutions but are available from the corresponding author on reasonable request.

## Supplementary Materials

The following supporting information can be downloaded at: https://www.mdpi.com/article/10.3390/genes15060713/s1; Table S1: Differentially expressed genes between M-AP and U animals; Table S2: Differentially expressed genes between H-AP and U animals; Table S3: Differentially expressed genes between AbMR-biased and U animals; Table S4: Differentially expressed genes between CMR-biased animals and U animals.
